# Rice Thematic Special Issue: Beneficial Plant–Microbe Interactions in Rice

**DOI:** 10.1186/s12284-023-00659-8

**Published:** 2023-11-03

**Authors:** Concha Domingo, Blanca San Segundo

**Affiliations:** 1 Departamento del Arroz and Centro de Genómica, Instituto Valenciano de Investigaciones Agrarias (IVIA), Ctra Moncada-Náquera km 10.7, 46113 Moncada, Valencia, Spain; 2 Centre for Research in Agricultural Genomics (CRAG) CSIC-IRTA-UAB-UB, Campus Universitat Autònoma de Barcelona (UAB), Bellaterra (Cerdanyola del Vallés), C/de la Vall Moronta, CRAG Building, 08193 Barcelona, Spain; 3 Consejo Superior de Investigaciones Científicas (CSIC), Barcelona, Spain

Plants associate with a large diversity of microorganisms, collectively known as the microbiome. Some of these microorganisms are capable of establishing symbiotic interactions with their host, and these beneficial plant–microbe interactions have enormous potential to improve plant growth and productivity under stressful environments. Beneficial microorganisms, like rhizobacteria and arbuscular mycorrhizal fungi, are generally recruited from the environment, while others reside in plant tissues (e.g. endophytes).

Due to constant changing climatic conditions and global warming, rice cultivation is facing urgent challenges. Agrochemicals (e.g. fertilizers, pesticides) are routinely used in rice cultivation to maintain optimal yield and to prevent losses caused by environmental stresses. However, the indiscriminate use of agrochemicals has adverse effects on human health and the environment. An overreliance on agrochemicals might be also detrimental to plant-associated microbial communities. Under this scenario, understanding and harnessing the association of rice with beneficial microorganisms might help to meet the increasing demand on rice production with reduced environmental impact. Interactions with different microorganisms that are beneficial for rice plants have been described, which include endophytic bacteria and fungi, rhizobacteria, and arbuscular mycorrhizal fungi (AMF). To better exploit the potential of beneficial microorganisms in rice cultivation, a better knowledge of the mechanisms underlying these interactions is needed.

This special issue covers significant advances on rice-microbe interactions and address important questions such as: (1) the capability of certain microorganisms to mitigate the adverse impacts of abiotic stresses (salinity, drought, heavy metal toxicity), or resistance to pathogen infection, (2) the importance of the plant genotype in the association with root associated microorganisms, (3) changes occurring in the rhizosphere as a result of beneficial interactions with microorganisms, (4) the extent to which the root mycobiota affects rice yield under stress conditions, and (5) the genetic bases underlying the beneficial effects associated to plant microbe-interactions in rice. With great pleasure, we thank all the contributors for their timely response and contributions to this Special Issue.

To deepen how plant growth-promoting endophytic bacteria enhance plant growth and mitigate plant from abiotic stresses, in this issue, Kruasuwan et al. analysed the molecular interactions of endophytic *Streptomyces* mutually associated with rice plants displaying different susceptibility to salinity. In this system, *Streptomyces* sp. GKU 895 promotes growth and alleviates salt tolerance. They could observe that the presence of bacteria caused significant alterations in the expression of rice genes and their associated transcription factors, impacting various aspects of growth and development, photosynthesis, plant hormones, ROS scavenging, ion transport and balance, as well as plant–microbe interactions. These changes encompassed proteins associated with pathogenesis and symbiosis, further emphasizing the role of bacteria in shaping these fundamental biological processes (see Fig. 1).

**Fig. 1:**
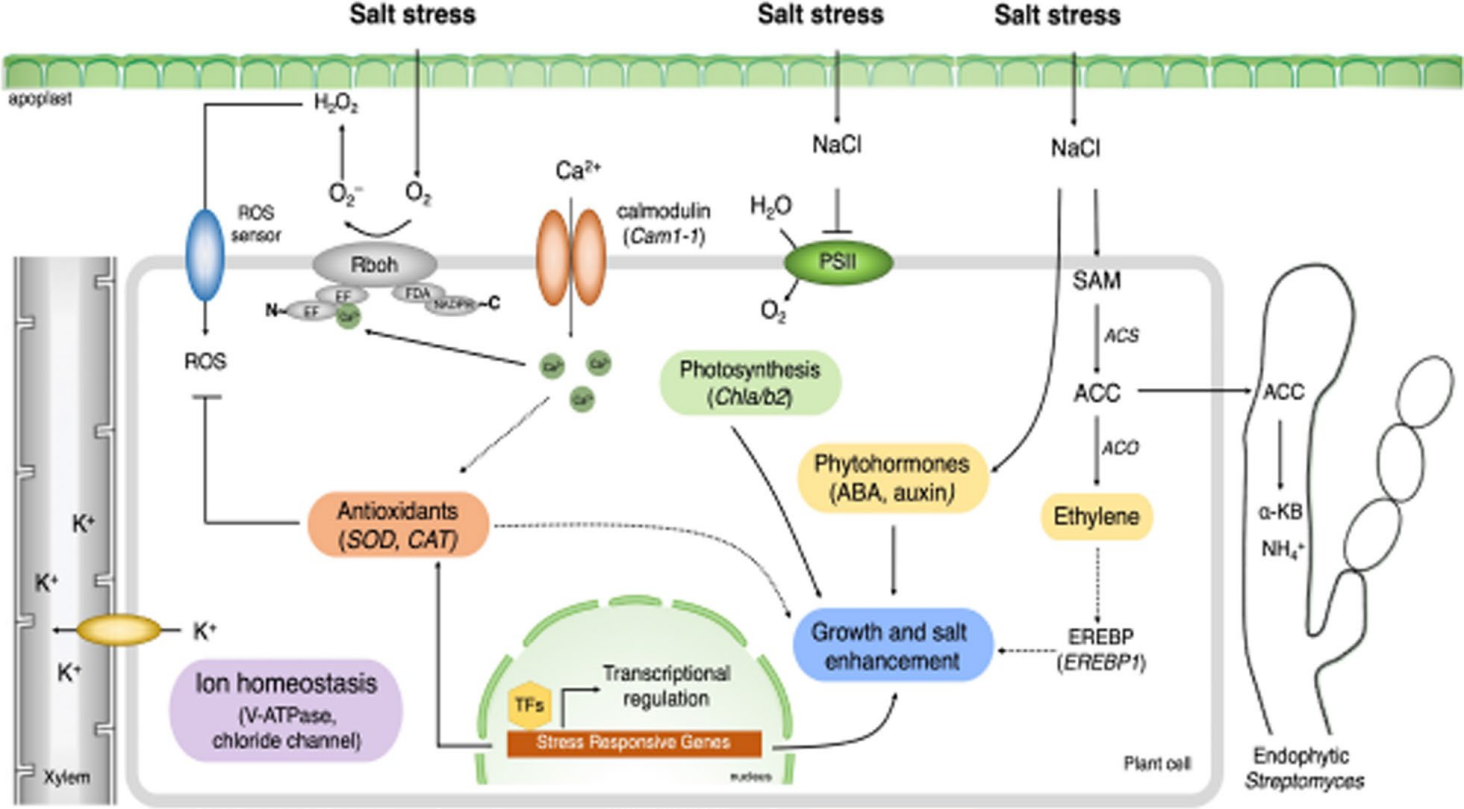
Mechanisms in the rice response to growth and salt tolerance conferred by plant growth-promoting endophytic *Streptomyces* sp. (Kruasuwan et al.)

To investigate to which extent the plant–microbe interaction is conditioned by plant genotype, Walitang et al. conducted an evaluation of protein profiles in two genetically related rice varieties following the inoculation of a versatile plant growth-promoting endophytic bacteria, *Methylobacterium oryzae* CBMB20. They discovered that despite approximately 90% of the proteins were similar in both genotypes, there were also unique responses specific to each genotype after inoculation with the bacterium. In the case of IR29, there was an observed increase in the abundance of proteins related to photosynthesis. On the other hand, FL478 exhibited a higher abundance of differentially abundant proteins associated with cell structure, differentiation, fate, as well as protein folding and transport. Additionally, the researchers noted that *M. oryzae* CBMB20 influenced the defense responses in both rice varieties.

Li et al. described changes in the rhizosphere microbiome and metabolite profiles of root exudates in two different rice cultivars and the effect of drought stress on the rhizosphere bacterial communities and root exudate composition. Integrated microbiome and metabolomic analyses showed that both genotype and drought had significant effects on the composition of root exudates and rhizosphere bacterial community, suggesting that the rice plant modify the rhizosphere microbiota via metabolites under stress (drought) conditions. A strong link was observed between various genera and organic acid and amino acid levels in root exudates which might be important for selection of specific rhizosphere bacterial communities under drought conditions. Although there are still issues that need to be addressed, evidence here presented will open new avenues for improving the adaptation of rice to drought stress from the perspective of interactions with beneficial microorganisms.

Mycorrhizal associations, particularly with AMF, have been extensively studied in rice. These fungi form mutualistic associations with rice roots, facilitating nutrient uptake, particularly phosphorus. AMF enhance the root surface area, improving nutrient absorption and water uptake in rice plants. Additionally, they contribute to stress tolerance, assisting rice plants in coping with drought, salinity, and other environmental challenges. Incorporating AMF in rice production systems can enhance nutrient efficiency, reduce fertilizer usage, and promote sustainable agriculture practices. In particular, high salinity is a major stress factor that seriously affect rice growth, development and yield. Studies carried out by Zhang et al. addressed the effects of AMF compound inoculants on growth, ion homeostasis and salt-tolerance-related gene expression in rice under salt stress conditions. The authors assessed the effects of co-inoculation of an AMF, *Piriformospora indica* and *Agrobacterium tumefaciens*, and concluded that AMF compound inoculants can improve the adaptability of rice under salt stress conditions and promote plant growth by regulating the photosynthetic intensity, ROS scavenging ability and ion homeostasis in rice plants.

Andreo-Jimenez et al. investigated several key aspects regarding the association between the plant host and root-associated microorganisms. They addressed aspects related to the role of the plant host in these associations, to investigate whether there are changes in microorganism recruitment during drought, and the extent to which the root mycobiota influences rice yield under both well-watered and drought conditions. Genetic loci associated with yield and the interaction with root-associated fungal microorganisms in rice plants were described.

Sehar et al. described the transcriptomic analysis of two rice genotypes differing in their responses to arsenic (i.e. low and high arsenic accumulators). The tolerance mechanisms of these genotypes upon exposure to arsenic under different phosphorus supply conditions and application of *Serendipita indica*, a root endophytic fungus, were investigated. The resistant rice genotype was found to have a higher content of auxin, which helped in minimizing the accumulation of arsenic and responding to phosphorus starvation. In contrast, the higher arsenic accumulating genotype displayed up-regulation of genes involved in ethylene signaling and programmed cell death under arsenic toxicity. In addition to genes involved in phytohormonal metabolism, differential expression of phosphate transporter genes and genes involved in protection against oxidative stress or metal detoxification/transport proteins potentially contributing to arsenic detoxification were identified. Overall, results obtained in this study support that the *S. indica* symbiosis, fortified with adequate P-fertilizer, is effective in minimizing arsenic accumulation in rice plants.

In conclusion, beneficial plant–microbe interactions offer immense potential for sustainable rice cultivation. Harnessing the capabilities of endophytic bacteria, rhizobacteria, mycorrhizal associations, nitrogen-fixing bacteria, and disease-suppressive microbes can improve nutrient uptake, reduce chemical inputs, enhance stress tolerance, and promote plant health in rice fields. Further research and practical application of these interactions hold the key to achieving sustainable rice production systems that are both environmentally friendly and economically viable.

## Data Availability

All data are included in the article.

## Abbreviation

AMF: Arbuscular mycorrhizal fungi
